# Enhanced secretion of an *Agrocybe aegerita* peroxygenase variant in *K. phaffii* using the native signal peptide

**DOI:** 10.1186/s13568-026-02049-x

**Published:** 2026-04-01

**Authors:** Ginevra Camboni, Rebecca Preece, Katy A. S. Cornish, Jared Cartwright, Gideon Grogan

**Affiliations:** 1 Departments of Chemistry, University of York, Heslington, YO10 5DD York, United Kingdom; 2 Departments of Biology, University of York, Heslington, York, YO10 5DD United Kingdom

**Keywords:** Unspecific peroxygenase, *Pichia pastoris*, *Komagataella phaffii*, *Saccharomyces cerevisiae*, Signal peptide

## Abstract

Unspecific Peroxygenases (UPOs) are enzymes with significant potential as catalysts in synthetic chemistry as they catalyse selective oxygenation reactions on organic substrates at the expense of only hydrogen peroxide as the external oxidant. The demand for UPOs has stimulated considerable research into efficient heterologous expression systems for their production, which have included optimisation of the signal peptide (SP) included upstream of the UPO gene. In this study we report a comparison of the production of the prototypical and widely-used UPO variant from the fungus *Agrocybe aegerita* (r*Aae*UPO-PaDa-I-H) using both its native SP and variant SPs evolved for improved heterologous expression in yeast hosts. The results show that improvements in protein production identified using variant SPs in *S. cerevisiae* are not necessarily extended to the yeast *Komagataella phaffii*. Indeed in *K. phaffii* we observed a five to sevenfold improvement in the activity of crude secretates produced using the native fungal SP of *Aae*UPO, compared with SPs that were evolved for improved expression and screened in *S. cerevisiae* These findings suggest that superior yields of this widely-used UPO can be obtained from scaled production in *K. phaffii* by using the native SP from the source fungus.

**Supplementary Information:**

The online version contains supplementary material available at 10.1186/s13568-026-02049-x.

## Introduction

Unspecific Peroxygenases (UPOs) are heme oxygenases that are secreted from fungi (Wang et al. 2017; Hobisch et al. 2021; Monterrey et al. 2023). Since their discovery in 2004 by Hofrichter and co-workers (Ullrich et al. 2004), they have been the focus of intense research for their potential as biocatalysts with applications in synthetic organic chemistry, as they are capable of the regio- and enantioselective oxygenation of a range of organic substrates in the mode of the more well-characterised heme oxygenases cytochromes P450 (P450s) (Grogan 2021; Schröder et al. 2023). UPOs display several advantages over P450s with respect to preparative biocatalysis however, as they depend only upon hydrogen peroxide as the external oxidant to generate the catalytic species Compound I in the heme active site (Wang et al. 2015) instead of the expensive nicotinamide cofactor (NADPH) and electron transfer proteins (P450 reductases or ferredoxin plus ferredoxin reductases) that are required by P450s to generate the same intermediate. In addition, UPOs can be easily fermented at scale in heterologous systems such as *Komagataella phaffii* (*Pichia pastoris*), secreted into the fermentation medium, and lyophilised for storage, obviating the need for microbial systems in organic synthesis laboratories where the enzymes will be applied. The first enzyme to be described, from the fungus *Agrocybe aegerita* (*Aae*UPO) has in this mode been successfully expressed in both *S. cerevisiae* (Molina-Espeja et al. 2014)and *K. phaffii* (*Pichia pastoris*) (Molina-Espeja et al. 2015) and fermentative production of an improved variant of greater activity, *Aae*UPO-PaDa-I (Molina-Espeja et al. 2014, 2015), has been scaled to 2500 L (Tonin et al. 2021). *Aae*UPO-PaDa-I has subsequently been applied in a host of useful oxygenation reactions (Wang et al. 2017; Hobisch et al. 2021; Monterrey et al. 2023; Hofrichter and Ullrich 2014; Bormann et al. 2015; Han et al. 2024; Pogrányi et al. 2023; Li et al. 2025), demonstrating its promise for synthetic applications.

One crucial element with respect to the heterologous expression of proteins in yeasts is the choice of signal peptide (SP), a short sequence of amino acids that is encoded upstream of the UPO gene and which directs the gene product to the endoplasmic reticulum (Owji et al. 2018; Nilsson et al. 2015) and secretion. In pioneering studies by Alcalde and co-workers (Molina-Espeja et al. 2014), directed evolution was used to generate the superior *Aae*UPO variant, *Aae*UPO-PaDa-I, with improved activity and expression levels in *S. cerevisiae*. The improvements in activity were attributed to nine point mutations in the amino acid sequence. Five of these were found in the mature enzyme, but four (F12Y/A14V/R15G/A21D) were in the SP, and reportedly improved secretion 27-fold in *S. cerevisiae*. A construct with all of these SP and enzyme sequence mutations was then successfully transferred to *K. phaffii* (Molina-Espeja et al. 2015), giving a strain that could be used for the large-scale fermentation mentioned previously (Tonin et al. 2021). In more recent work, Weissenborn and co-workers applied the Golden Gate^®^ system to enable the shuffling of SPs with UPO genes, revealing that, in some cases, superior heterologous expression of a UPO could be achieved using a non-cognate SP (Püllmann et al. 2021; Dietz et al. 2023). In the interests of developing a high-throughput screen for improved gene expression in yeast systems using evolved SPs, we recently described a reporter system based on the activity of the luciferase from *Gaussia princeps* (Camboni et al. 2025). Using truncated variant *Aae*UPO constructs as model systems we showed that SP variants generated using error-prone PCR could be screened in a high-throughput manner, in the process identifying a new SP variant for improved *Aae*UPO protein production, 1C1-SP, containing mutation Y22N, which had not been reported previously. We also showed that 1C1-SP was able to improve protein production for a full-length r*Aae*UPO-PaDa-I-H in *S. cerevisiae* against established constructs that employed the native SP (n-SP) and PaDa-I variant SP (PaDa-I-SP).

In this study we investigated whether the variant SPs that had been evolved for improved protein production in *S. cerevisiae* also enabled equivalent improvement in the enzyme production strain *K. phaffii*. We selected the 1C1-SP variant from our previous experiment (Camboni et al. 2025) and tested its functionality in *K. phaffii* alongside n-SP and PaDa-I-SP. Transposing the SPs from *S. cerevisiae* to *K. phaffii* for protein production showed that the SP that was optimal for protein production in *S. cerevisiae* was not optimal for *K. phaffii*; indeed, superior amount of enzyme were produced using the n-SP from the source fungus *A. aegerita*. These results demonstrate the importance of identifying a balance between the SP, the target enzyme, and the host organism for optimal secretion of target proteins.

## Materials and methods

### Restriction enzymes

Restriction enzymes were purchased from New England Biolabs (Ipswich, UK).

### Cells and cultivation media

*E. coli* Stellar competent cells were purchased from Takara Bio Europe Clontech (St Germain-en-Laye, France). *K. phaffii* X-33 strain was purchased from Invitrogen (ThermoFisher, UK). For *E. coli* growth, LB was purchased from Merck Chemicals Ltd. (Nottingham, UK). For *K. phaffii* growth, Yeast Extract Peptone (YEP) was purchased from Merck Chemicals Ltd. (Nottingham, UK). Yeast nitrogen base (YNB) was obtained from Sigma-Aldrich Company Ltd. (Dorset, UK).

### Oligonucleotides and genes

Oligonucleotides were purchased in the lowest purification grade “desalted” and minimal quantity from IDT Integrated DNA Technologies (Leuven, Belgium). *Aae*UPO variant genes were previously generated in our laboratory (Camboni et al. 2025).

### Expression plasmid construction for *K. phaffii*

The vector pPICZB (invitrogen) was used as the backbone for expression in *K. phaffii* and for propagation in *E. coli*. The vector was previously constructed by Dr. Tamara Mielke in our laboratory for the production of r*Aae*UPO-PaDa-I-H under the control of the PaDa-I SP (PaDa-I-SP/r*Aae*UPO-PaDa-I-H in this manuscript; Bonfield et al. 2020).

To create the 1C1-SP/r*Aae*UPO-PaDa-I-H and n-SP/r*Aae*UPO-PaDa-I-H constructs, the plasmid pPICZB/PaDa-I-SP/r*Aae*UPO-PaDa-I-H was linearized by inverse PCR to delete the PaDa-I SP. The signal peptides n-SP and 1C1-SP were amplified by High-Fidelity PCR using KOD polymerase (Merck), following the manufacturer’s instructions. The linear vector was used in In-Fusion cloning with the SP sequences n-SP or 1C1-SP in a 2:1 ratio, respectively. In-Fusion was carried out at 50 °C for 15 min and the products were used to transform *E. coli* Stellar competent cells (TakaraBio) for plasmid propagation. *E. coli* cells were plated onto agar containing low-salt Luria Broth (LB) and zeocin (25 µg mL^−1^) and incubated for 16 h at 37 °C. The following day, colonies were picked and inoculated into 10 mL of LB containing zeocin (25 µg mL^−1^). After 16 h growth, plasmids were purified using a QIAgen miniprep kit. The purified plasmids were sequenced by Eurofins Genomics, confirming the respective SP in the constructs pPICZB/1C1-SP/r*Aae*UPO-PaDa-I-H, pPICZB/n-SP/r*Aae*UPO-PaDa-I-H and pPICZB/PaDa-I-SP/r*Aae*UPO-PaDa-I-H.

### Transformation of *K. phaffii*

*K. phaffii* transformation was performed by integrating the linear DNA template into the genome of the organism. Plasmids were linearized in a vial containing: 2 µL of 10X Cutsmart buffer, 5 U µL^−1^ of *PmeI* restriction enzyme, 1 µg of DNA template and nuclease-free water to a total volume of 20 µL. Digestion was carried out for 1 h at 37 °C. The product was run on a 1% agarose gel, purified with a QIAgen gel extraction kit, and eluted in 30 µL of nuclease-free water. The template concentration obtained was routinely between 5 and 20 ng µL^−1^.

*K. phaffii* strain X-33 was streaked on a YPD plate and incubated at 30 °C for 2–3 d. One colony was inoculated in 100 mL of YPD in a 500 mL flask and grown overnight at 30 °C with shaking at 180 rpm. The following day, when the OD_600_ was measured to be a value between 1 and 2, the cells were centrifuged at 3000×*g* for 5 min at 4 °C. They were then resuspended in 20 mL of buffer containing 0.6 M sorbitol, 0.1 M lithium acetate and 10 mM Tris-HCl at pH 7.5. 250 µL of 1 M DTT was added to the cells, and the tube was gently inverted to mix. The cells were then incubated at room temperature for 20–30 min and the tubes were inverted occasionally to prevent sedimentation. The cells were then washed by centrifuging at 3000×*g* for 5 min at 4 °C, re-suspended in 25 mL of ice cold 1 M sorbitol, and incubated on ice for 5 min. The washing step was repeated twice, and following the final centrifugation, the pellet was re-suspended in 500 µL of ice-cold 1 M sorbitol in order to obtain a final 1–2 mL of thick cell suspension. 80 µL of this was transferred to an ice cold 2 mm electroporation cuvette. Between 100 and 200 ng of linear DNA was added to the cuvette and the cells were incubated on ice for 5 min, along with two 2 mL tubes and a recovering solution of 50% 1 M Sorbitol 50% YPD. Electroporation was carried out by running one pulse using a Biorad gene pulser at 1.5 kV, a capacitance of 25 µF, and a resistance of 400 Ω. Immediately after electroporation, 1 mL of the ice-cold recovering solution was added to the cuvette, mixed with the sample by pipetting and the cell suspension was transferred to the 2 mL tube. The cells were incubated at 30 °C for 4 h at 600 rpm to prevent sedimentation. After the incubation time, 100 µL of the cell suspension was plated on YPD zeocin (100 µg µL^−1^) plates, and incubated at 30 °C for 3 d. Following the incubation time, 4 colonies each were re-streaked again on fresh YPD zeocin plates and incubated at 30 °C for 3 d.

### *K. phaffii* colony PCR

One colony was picked with a 10 µL tip and transferred in a 250 µL tube containing 30 µL of 20 mM NaOH. The tip was washed in the solution, creating a cloudy mixture. The samples were boiled at 100 °C for 20 min. The tubes were then centrifuged for 30 s, and between 1 and 2 µL of supernatant was used as template in the PCR mix for amplification. PCR was performed with Q5 polymerase, following the manufacturer instructions, and the PCR product was routinely purified with a QIAgen PCR purification kit. Sequencing was performed by Eurofins Genomics.

### *K. phaffii* small scale expression

Four colonies of each *K. phaffii* transformant were tested for protein production. For the experiment, stock solutions were prepared according to the ‘EasySelect Pichia expression kit’ manual (Invitrogen), which were then used to prepare BMGY and BMMY media solutions (recipes are given in the Supplemental File). Four colonies of *K. phaffii* transformants were used to inoculate 5 mL of BMGY each. The cultures were incubated for 20 h at 30 °C with shaking at 230 rpm. The following day, samples t = 0 were collected and 250 µL of each starting culture was inoculated in 5 mL of BMMY. The cultures were incubated at 30 °C, with shaking at 230 rpm for the remainder of the expression experiment. The remaining 5 mL of BMMY were used as feeding solution: 4.5 mL was removed from the medium and 500 µL of 100% methanol was added to give 5 mL of BMMY 10% methanol.

Every 24 h until 96–120 h, 250 µL samples were collected and 250 µL of BMMY 10% methanol feeding solution was added to the culture. Each sample was centrifuged at 13,000×*g* for 5 min, the supernatant was collected in a 1.5 mL tube and then stored at − 20 °C prior to protein production analysis.

### Fermentation in 200 mL bioreactor

A 200 mL minimal medium solution, PTM_1_ salts solution, a glycerol feed solution (50% w/v glycerol containing 12 mL per Liter PTM_1_ salts) and methanol feed solution (100% methanol containing 12 mL L^−1^ PTM_1_ salts) was prepared, according to instructions in the “Pichia Fermentation Process Guidelines” manual (Invitrogen).

Two colonies of each variant were used to inoculate Yeast extract, peptone and glucose medium (YPD) and incubated at 30 °C for 24 h, at 230 rpm. When the OD_600_ reached 30, the cultures were used to inoculate the vessel of the bioreactor, where the temperature was set to 30 °C and the pH was kept constant at 4.75. The first glycerol batch phase lasted 24 h, then, when a spike in DO (dissolved oxygen) was observed, the glycerol fed-batch was initiated at a rate of 0.01 mL min^−1^, and was continued for 4 h. A total volume of 15 mL of glycerol was consumed by each culture, and when a second spike in DO was observed, the temperature was decreased to 25 °C, the methanol fed-batch was initiated at a rate of 3.6 mL h^−1^ L^−1^ and then kept constant overnight. Samples were collected at t = 0 (before the methanol fed-batch was initiated), at t = 24 h, t = 48 h and t = 72 h after induction. 2 mL samples were collected and divided in two 1.5 mL tubes, containing 1 mL of sample in each. The tubes were centrifuged at 13,000×*g* for 1 min, the supernatant removed and stored at − 20 °C for protein production analysis. The wet-cell weight was then determined. After 72 h of methanol fed-batch growth the cultures were harvested and centrifuged at 5000×*g* for 10 min. The supernatants of each culture were recovered for storage at − 80 °C prior to protein purification.

### Western blot

SDS-PAGE 12% gels were run with the supernatant samples collected at time intervals during protein production, using as a marker PageRuler™ Plus Prestained Protein Ladder (10 to 250 kDa; ThermoFisher Scientific, UK). Protein transfer was performed with Invitrogen iBlot 2 dry blotting system set at 20 V and 1 Amp for 6 min. The nitrocellulose membrane was washed with blocking buffer PBST 5% milk (PBS, Tween-20 0.1%, 5% milk) and then blocked for 3 h with gentle shaking at room temperature. The membrane was then incubated overnight in blocking buffer containing 1:3000 Anti-his Ab (Produced in mouse, Sigma). Following overnight incubation, the membrane was washed repeatedly with PBS buffer, and then visualized on iBright Invitrogen imager following 5 min incubation at RT with ECL (Invitrogen) chemiluminescent solution.

#### Protein purification

Ammonium sulfate precipitation was first performed by slowly adding ammonium sulfate to the secretates to a final concentration of 40% saturation. Following centrifugation at 10,000×*g*, the supernatant was then applied to a 5 mL HiTrap Phenyl Sepharose column (Cytiva) equilibrated in 20 mM Tris-HCl, pH 8.0, 1.5 M ammonium sulphate (Buffer A). Following removal of non-specific binding using 10 column volumes (CV) of Buffer A, specific protein was eluted with a gradient from 40 to 0% ammonium sulfate applied over 15 CV. Eluting peaks were monitored for absorbance at both 280 nm and 420 nm. The fractions determined to contain the enzyme by SDS-PAGE were pooled, dialysed against a low salt buffer (20 mM Tris-HCl, pH 8.0) and applied to a 5 mL Bioscale Q, Biorad) anion-exchange column, equilibrated in 20 mM Tris-HCl, pH 8.0 (Buffer B). Following removal of non-specific binding using 5 CV of Buffer B, bound protein was eluted with 0 to 300 mM NaCl gradient over 10 CV, then rapidly increased to 2 M for a further 4 CV. Elution was monitored at absorbances of 280 nm and 420 nm. Despite the enzyme r*Aae*UPO-PaDa-I-H binding initially to the column, the protein eluted in a sharp peak during the column wash of the anion exchange resin, indicating weak binding at pH 8.0. Higher pH value buffers were not assessed as the contaminant proteins remained largely bound to the resin and eluted during the NaCl gradient step, overall permitting effective fractionation of the target protein. The wash-fraction was pooled, concentrated and applied to a Hiload 16/600 Superdex S200 pg (Cytiva) size exclusion chromatography column, equilibrated in 20 mM Tris-HCl pH 8, 150 mM NaCl (Buffer C). Isocratic elution was performed with 1.2 CV of Buffer C and fractions of interest were collected based on A280 nm and A420 nm absorbance. The pooled fractions were further analysed for purity by SDS-PAGE and by UV spectrophotometry to determine protein concentration before storage at − 70 °C.

### UV/Vis activity assays

ABTS assay: The reaction screening was performed using a 1 mL UV quartz cuvette (path length 1 cm) containing 50 mM citrate phosphate buffer (Na_2_HPO_4_ 200 mM, citric acid 100 mM buffer), pH 4.4, 0.2 mM ABTS, 2–5 µL of crude supernatant sample and 2 mM H_2_O_2_. The absorbance was measured at 418 nm over a period of 1 min. To measure activity (U in µmol min^−1^), the change in absorbance was plotted against time and the reaction rate (*k*_obs_) was determined using the ABTS molar extinction coefficient (ε_418_) of 36,000 M^−1^ cm^−1^.

NBD assay: The reaction screening was performed using a 1 mL UV quartz cuvette (path length 1 cm) containing KPi buffer 50 mM, pH 7.0, 1 mM NBD, 1–5 µL of crude supernatant sample and 2 mM H_2_O_2_. The absorbance was measured at 420 nm over a period of 2 min. To measure activity (U in µmol min^−1^), the change in absorbance was plotted against time and the reaction rate (*k*_obs_) was determined using the NBD extinction coefficient (ε_420_) of 9700 M^−1^ cm^−1^.

## Results

**Fig. 1:**
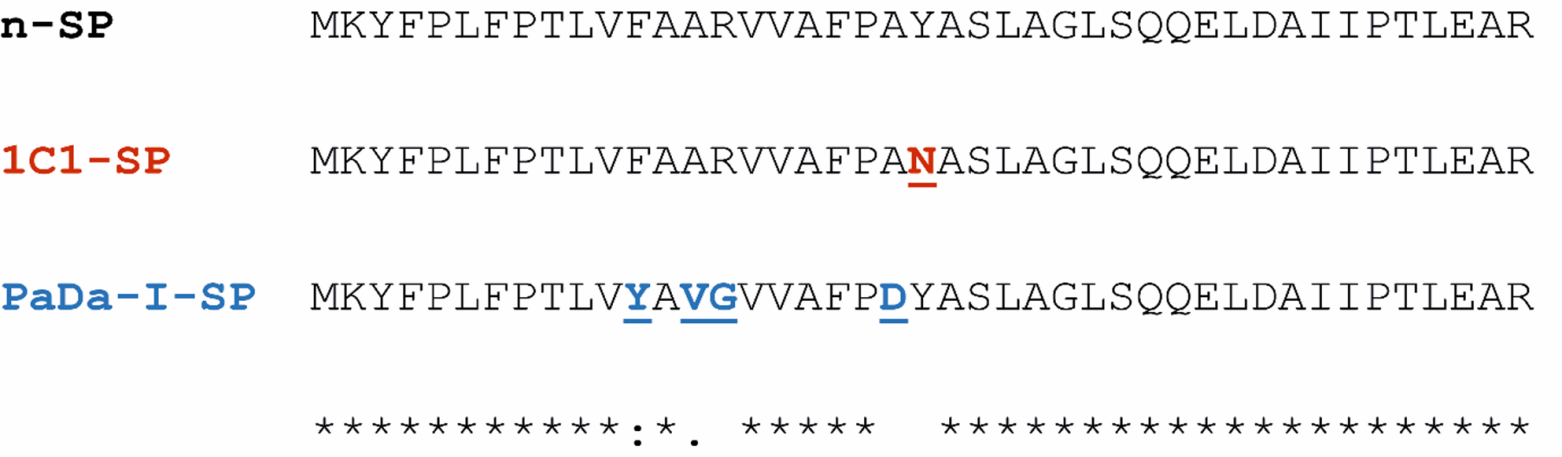
Sequence alignment of n-SP, 1C1-SP and PaDa-I-SP, with their mutations highlighted in red and blue, respectively. Each SP was fused upstream of the r*Aae*UPO-PaDa-I-H gene, which contains five mutations from the wild-type *Aae*UPO enzyme sequence: The 1C1-SP has mutation Y22N; PaDa-I-SP has mutations: F12Y, A14V, R15G and A21D

In our previous report (Camboni et al. 2025) we described a universal method for the screening of SP variants produced using error-prone PCR, employing an assay based on the activity of *Gaussia* luciferase (GLuc). From this study we identified the SP variant 1C1-SP which, when tested in *S. cerevisiae* for the expression of full-length variant r-*Aae*UPO-PaDa-I-H, enabled 13-fold improved secretion of the target protein against those produced using both the n-SP and PaDa-I SP sequences. In order to test whether this variant SP would also confer superior enzyme production in the favoured production organism *K. phaffii*, we undertook to introduce the 1C1-SP/r*Aae*UPO-PaDa-I-H construct into *K. phaffii*, in parallel with constructs containing the native SP (n-SP/r*Aae*UPO-PaDa-I-H) and the PaDa-I-SP (PaDa-I-SP/r*Aae*UPO-PaDa-I-H), in order to compare performance. The amino acid sequences of the three SPs (n-SP, 1C1-SP and PaDa-I-SP) are shown in Fig. 1.

### *K. phaffii* constructs

The vectors for production of the r*Aae*UPO-PaDa-I-H protein from *K. phaffii* were developed using the pPICZB vector with the *Aae*UPO 5-point mutant PaDa-I genetic sequence downstream of the PaDa-I signal peptide (pPICZB//PaDa-I-SP/r*Aae*UPO-PaDa-I-H) (Moline-Espeja et al. 2015; Bonfield et al. 2020). This vector contained the whole r*Aae*UPO-PaDa-I-H genetic sequence, including a 3 C site, a linker region and a His_6_ tag at the C-terminus of the protein (hence ‘r*Aae*UPO-PaDa-I-H’), for the purpose of immunoblot analysis. The pPICZB vector is a plasmid that contains the AOX1 promoter for the production of proteins, which is tightly regulated by methanol. The pPICZB vector also contains the zeocin resistance gene, for the selection of transformants from *E. coli* (for plasmid recovery) and from *K. phaffii*. The vectors with the 1C1-SP and the native *Aae*UPO-SP were named pPICZB/1C1-SP/r*Aae*UPO-PaDa-I-H and pPICZB/n-SP/r*Aae*UPO-PaDa-I-H, respectively.

**Fig. 2:**
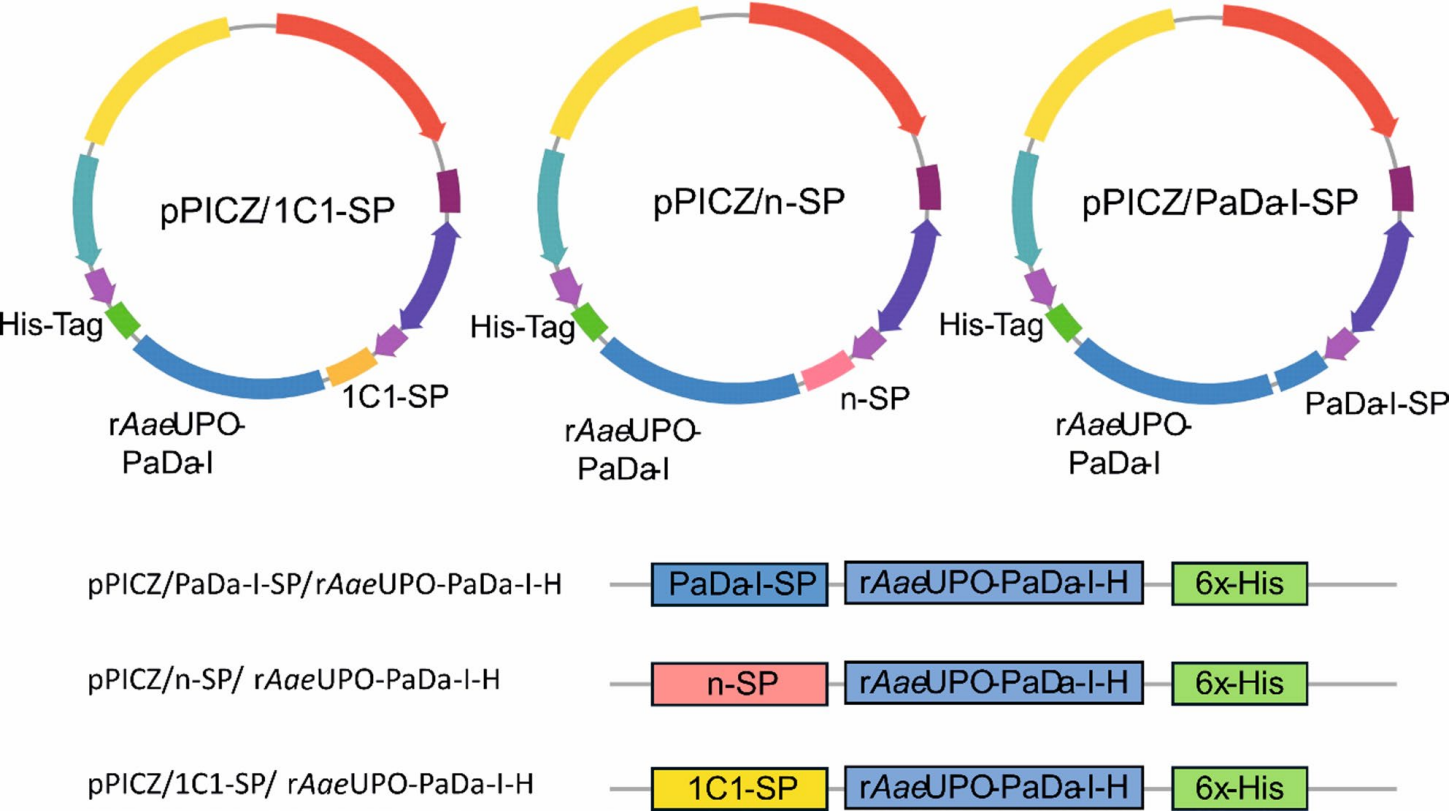
Constructs developed for production of r*Aae*UPO-PaDa-I-H in *K. phaffii*, under the PaDa-I (pPICZ/PaDa-I-SP/r*Aae*UPO-PaDa-I-H), the native (pPICZ/n-SP/r*Aae*UPO-PaDa-I-H) and the 1C1 (pPICZ/1C1-SP/r*Aae*UPO-PaDa-I-H) SPs

The native *Aae*UPO SP (n-SP) and the mutant 1C1-SP (1C1-SP) were amplified by PCR as described in the Experimental Section and the vector pPICZB/r*Aae*UPO-PaDa-I-H was linearized by inverse PCR to delete the PaDa-I-SP. In-Fusion cloning was performed and four colonies of each construct were selected for DNA purification and sequencing, which validated the clones pPICZB/1C1-SP/r*Aae*UPO-PaDa-I-H and pPICZB/n-SP/ r*Aae*UPO-PaDa-I-H (Fig. 2).

The plasmids pPICZ/PaDa-I-SP/ r*Aae*UPO-PaDa-I-H), pPICZ/n-SP/ r*Aae*UPO-PaDa-I-H and pPICZ/1C1-SP/ r*Aae*UPO-PaDa-I-H were initially recovered from *E. coli* and used for transformation of *K. phaffii* strain X-33 through integration of the linear vectors by electroporation. Colony PCR was carried out on four colonies of each of the strains transformed with the three vectors and sequencing of DNA of the transformants again validated the presence of the r*Aae*UPO-PaDa-I-H gene adjacent to the corresponding SPs.

### Small scale gene expression in *K. phaffii*

**Fig. 3:**
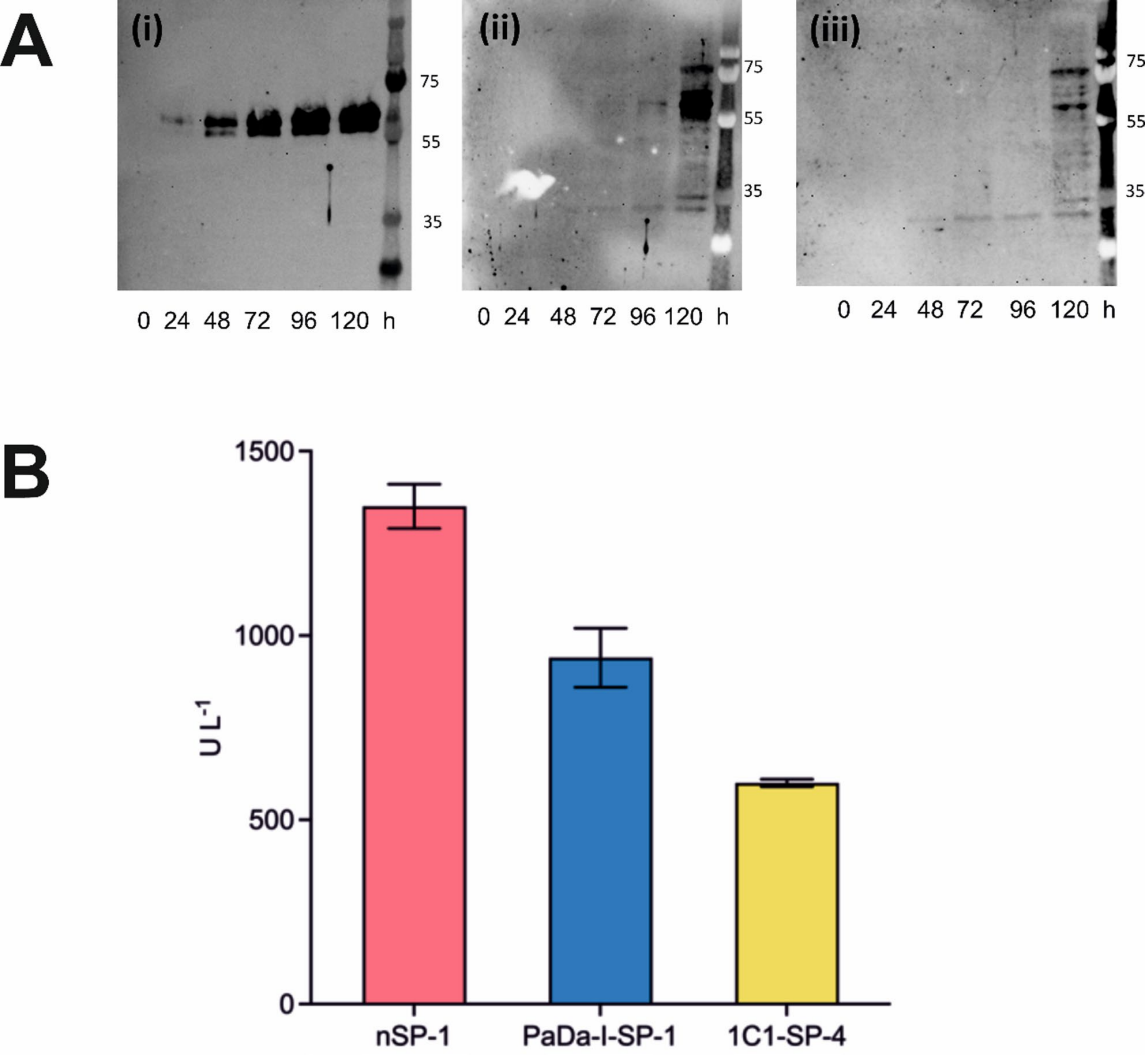
Preliminary small-scale tests. **A** Time course Western blot analysis of the the best clones from four analysed for each SP construct: (**i**) Sample 1 from n-SP/r*Aae*UPO-PaDa-I-H; (**ii**) Sample 1 from PaDa-I-SP/r*Aae*UPO-PaDa-I-H; (**iii**) Sample 4 from 1C1-SP/r*Aae*UPO-PaDa-I-H; **B** NBD assay of secretate extracted after 120 h protein production. Three technical replicates were performed on the same samples used for the blot analysis in **A**

Preliminary protein production experiments were initially performed on a small scale (5 mL) for the purpose of identifying the best clone from which the highest yield of protein could be produced (Materials and Methods Section). Protein production levels were initially assayed by Western blots, using antibody raised against the His-tag fused onto r*Aae*UPO-PaDa-I-H. The expected size of the r*Aae*UPO-PaDa-I-H from *K. phaffii* was estimated to be between 55 and 60 kDa, larger than the MW predicted from sequence, owing to extensive glycosylation of the protein. The best clones from four analysed for each SP construct (samples no. 1, each from PaDa-I-SP/r*Aae*UPO-PaDa-I-H and n-SP/r*Aae*UPO-PaDa-I-H and sample no. 4 from 1C1-SP/ r*Aae*UPO-PaDa-I-H), selected based on their highest band intensity from blot analysis (Fig. 3A), were retained for peroxygenase activity measurements using the well-established spectrophotometric assay based on the oxygenation of 5-nitro-1,3-benzodioxole (NBD) (Fig. 3B) (Dolz et al. 2023).

The Western blot analysis suggested that r*Aae*UPO-PaDa-I-H production under the native SP was more pronounced than with either of the evolved SPs. In addition, peroxygenase activity of the extracts of the n-SP cultures was higher (1350 ± 60 U L^−1^) than that of cultures transformed with constructs using either the PaDa-I-SP (940 ± 80 U L^−1^) or 1C1-SP (600 ± 10 U L^−1^) respectively.

**Fig. 4:**
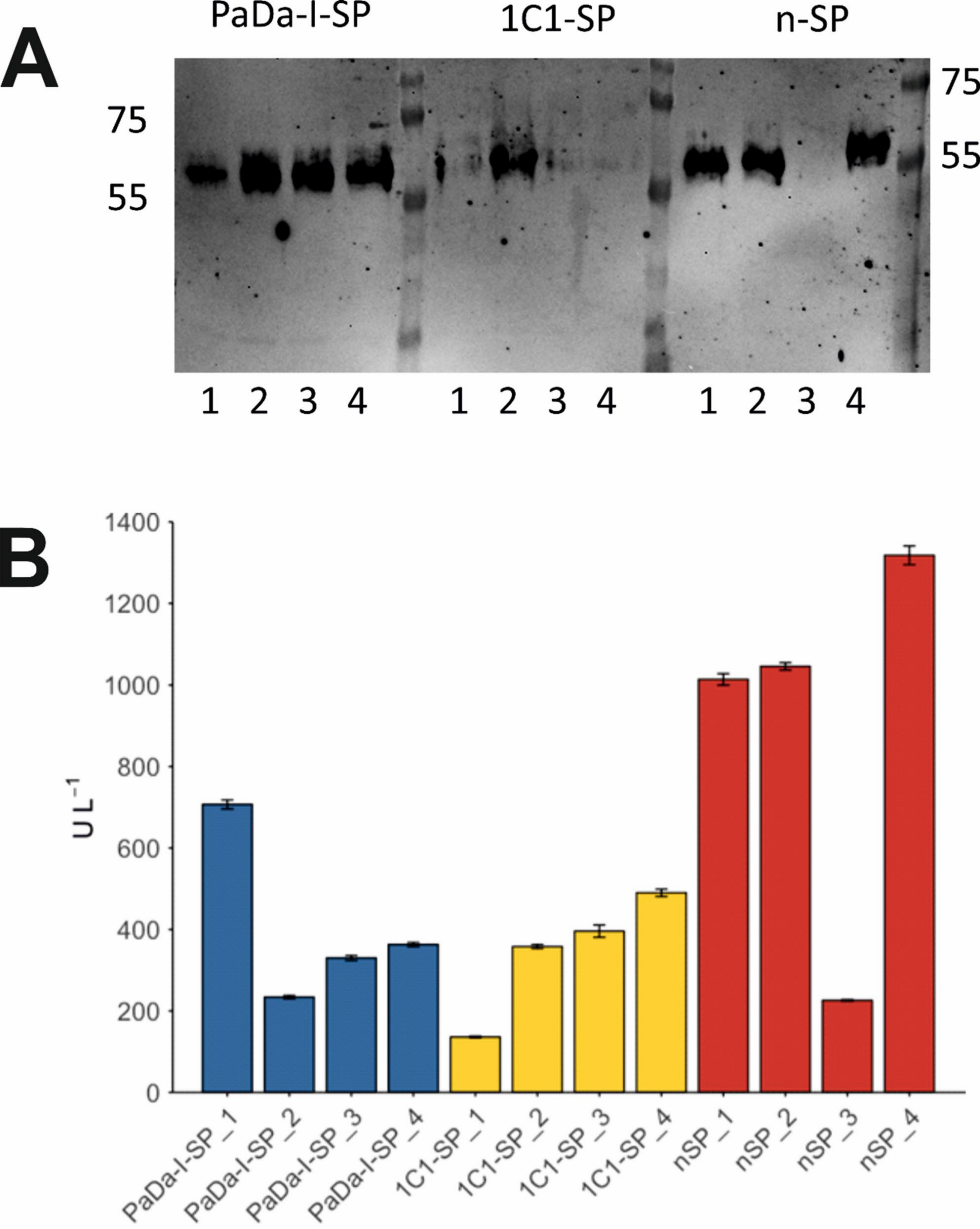
Second gene expression test. **A** Western blot of samples from clones 1–4 collected at 120 h (thus *n* = 4 biological replicates); **B** NBD assay performed on samples collected from clones 1–4 at 120 h

This unexpected observation prompted us to confirm the result with additional transformations of *K. phaffii*, in which new clones were selected. Successful integration of all three pPICZB constructs in the *K. phaffii* genome was again confirmed and verified by colony PCR and by sequencing. Protein production was repeated in 5 mL culture, with four transformants (1–4 in Fig. 4) each of cells using PaDa-I-SP/r*Aae*UPO-PaDa-I-H, 1C1-SP/r*Aae*UPO-PaDa-I-H and n-SP/r*Aae*UPO-PaDa-I-H (1–4) selected for Western blot analysis (Fig. 4A) and NBD assays (Fig. 4B).

In some cases, the NBD assay appeared to reveal higher activities for samples that gave fainter bands in the blot, as with sample no. 1 from PaDa-I-SP/r*Aae*UPO-PaDa-I-H and sample no. 4 from 1C1-SP/ r*Aae*UPO-PaDa-I-H. A likely explanation for the discrepancy is cleavage of the His tag from constructs during the expression. For this reason, the NBD assays are likely to be a more reliable indication of protein production overall.

### 200 mL scale expression experiments

**Fig. 5:**
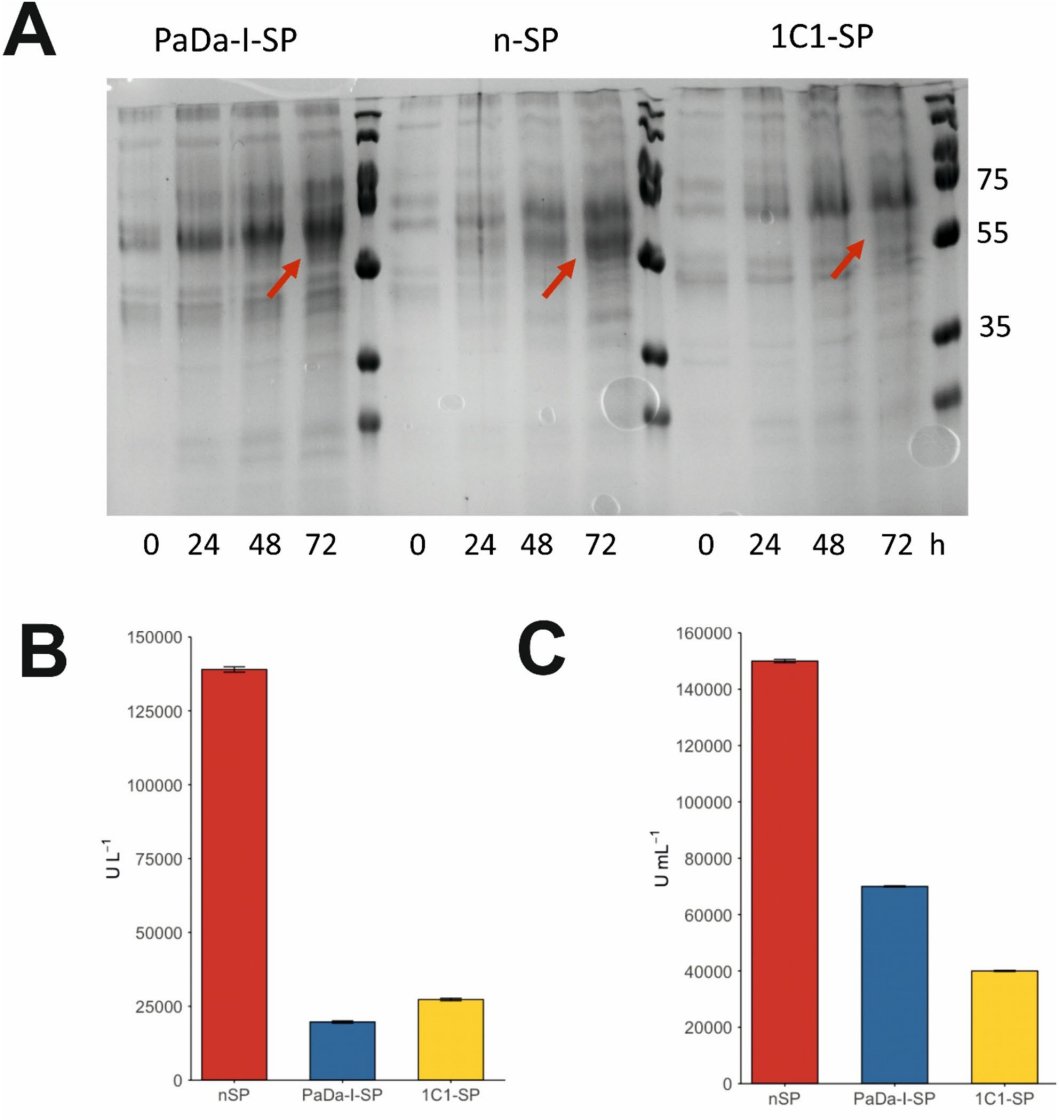
**A** SDS-PAGE time-course analysis of secretate samples collected during the 200 mL scale expressions in fermenters. The expected size of glycosylated r*Aae*UPO-PaDa-I-H is 55–60 kDa, indicated by a red arrow. An SDS-PAGE gel comparing a sample of the glycosylated enzyme and one that has been deglycosylated with Endo-H can be found in the Supplemental File. **B** NBD assay performed on the same samples collected at 72 h; **C** ABTS assay performed on the same samples collected at 72 h

Further investigations were conducted by producing r*Aae*UPO-PaDa-I-H by fermentation of *K. phaffii* in 200 mL bioreactors, with the final aim of comparing the yield of the protein obtained under the PaDa-I-SP, 1C1-SP and n-SPs through activity assays and protein purification. The colonies that gave cultures with the best activity towards NBD from the second small-scale expression test (Fig. 4) were selected for fermentation: sample no. 1 using PaDa-I-SP, and sample no. 4 each from those using 1C1-SP and n-SP. In an attempt to ensure consistency of method between the three fermentations, the three bioreactors were run simultaneously. Details of the fermentation procedure are given in the Experimental Section. Graphs describing dissolved oxygen, temperature, pH and other parameters can be found in the Supplemental File (Figure S6A–C), in addition to wet cell weight measurements at time intervals (Table S4). During fermentation, samples were collected at t = 0 h (before the methanol fed-batch phase was initiated) and at 24 h, 48 h and 72 h after induction. All three cultures displayed healthy growth until the end of the process, and at 72 h each culture was harvested. Secretates in each case were retained for protein production analysis using SDS-PAGE (Fig. 5A), the peroxygenase assay with NBD (Fig. 5B) and also the widely-used peroxidase activity assay using 2,2′-azino-bis(3-ethylbenzothiazoline-6-sulfonic acid (ABTS, Fig. 5C) (Dolz et al. 2023).

From the SDS-PAGE analysis (Fig. 5A) a band between 55 and 60 kDa, the expected size of r*Aae*UPO-PaDa-I-H, was observed for all samples, but with different intensities for 1C1-SP/r*Aae*UPO-PaDa-I-H, PaDa-I-SP/r*Aae*UPO-PaDa-I-H and n-SP/r*Aae*UPO-PaDa-I-H in increasing order. The results of the NBD assay (Fig. 5B) indicated 5-fold and 7-fold greater activity for extracts from fermentations conducted using n-SP/r*Aae*UPO-PaDa-I-H over those using 1C1-SP/r*Aae*UPO-PaDa-I-H and PaDa-I-SP/r*Aae*UPO-PaDa-I-H, respectively. Also, the ABTS assay (Fig. 5C) indicated 2.10-fold and 3.75-fold greater activity for protein obtained from fermentations using n-SP/r*Aae*UPO-PaDa-I-H than from those using PaDa-I-SP/r*Aae*UPO-PaDa-I-H and 1C1-SP/r*Aae*UPO-PaDa-I-H respectively.

### Purification of r*AaeUPO*-PaDa-I-H

**Fig. 6:**
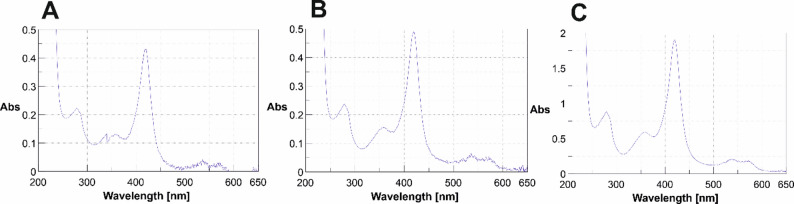
UV-Vis spectra of purified r*Aae*UPO-PaDa-I-H derived from fermentations using constructs **A** 1C1-SP- r*Aae*UPO-PaDa-I-H; **B** PaDa-I-SP- r*Aae*UPO-PaDa-I-H; **C** n-SP- r*Aae*UPO-PaDa-I-H

In order to obtain more data on the relative levels of protein production and activity of *Aae*UPO prepared using the different constructs, r*Aae*UPO-PaDa-I-H was purified from each fermentation, as described in the Materials and Methods Section. UV-Vis scans of the crude secretates obtained from each of the fermentations suggested that the peak at 418 nm, indicative of the heme iron bound to water, and which was absent in *K. phaffii* secretates expressing no r*Aae*UPO-PaDa-I-H, was more significant in the n-SP secretate, as was the Reinheitszahl (Rz) value (ratio of 418 nm to 280 nm) for this sample (0.12 vs. 0.10 for each of the other SPs). The relative enrichment of r*Aae*UPO-PaDa-I-H was also observed in the output of purification columns, including the first hydrophobic interaction column used in purification, and also in the final purified proteins from fermentations of each construct (Fig. 6A–C).

A comparison of Rz values of the crude secretates and purified proteins permitted an estimation the approximate yield of enzyme in each fermentation, giving values of 84.1 mg L^−1^, 89.1 mg L^−1^ and 365 mg L^−1^ from 1C1-SP/*Aae*UPO, PaDa-I-SP/r*Aae*UPO-PaDa-I-H and n-SP/r*Aae*UPO-PaDa-I-H respectively. Enzyme production using the n-SP/r*Aae*UPO-PaDa-I-H construct yielded 4.1-fold and 4.4-fold greater amounts of enzyme compared to PaDa-I-SP/r*Aae*UPO-PaDa-I-H and 1C1-SP/r*Aae*UPO-PaDa-I-H respectively therefore.

## Discussion

There are numerous examples of studies of the effect of different SPs on the expression of various gene targets in both *S. cerevisiae* (e.g. Xue et al. 2022) and *K. phaffii* (e.g. Barrero et al. 2018). Previous studies have also highlighted the importance of the SP in the successful heterologous expression of UPO genes in yeast systems (Molina-Espeja et al. 2014, 2015; Püllmann et al. 2021; Dietz et al. 2023). In this report, we undertook to examine whether an SP variant that improved the production of r*Aae*UPO-PaDa-I-H in *S. cerevisiae* would also serve to improve expression of the same target in *K. phaffii*. Interestingly, the SP (1C1-SP) identified from our previous directed evolution study (Camboni et al. 2025) that conferred the best protein production in *S. cerevisiae* was not the most effective in *K. phaffii*. Instead, it appears that using the native SP (n-SP) from the source fungus *A. aegerita* gave the best yield of r*Aae*UPO-PaDa-I-H.

It must first be considered that improved expression may to some extent be due to an increase in the copy number of the r*Aae*UPO-PaDa-I-H gene in the superior clones carrying the n-SP (Vogl et al. 2018). Multiple gene integrations into the host chromosome can be used as a tool for increased protein production in *K. phaffii* (Cesca Piva et al. 2017) and protein production has in many cases been correlated with higher copy number (Mansur et al. 2005; Liu et al. 2023), although in some cases this has also led to decreased protein production through overloading of the secretory pathway (Inan et al. 2006). However, for each of the experiments herein, comparing protein production using the different SPs described, equivalent procedures were used and of four biological replicates in the second screen (Fig. 4) three cases demonstrated higher levels of protein production for the n-SP construct on average. Additionally, no strategies for deliberately increasing copy number in *K. phaffii*, such as the introduction of new recombination systems (Li et al. 2017) or post-transformational vector amplification (Aw and Polizzi 2016), were applied in these experiments. However, the number of gene copies in the strains of *K. phaffii* bearing the constructs with different SPs may in future be clarified through the use of quantitative techniques such as RT-PCR, which have previously been applied for this purpose in studies of protein production using this host (Abad et al. 2010).

Reports that compare the relative performance of a range of UPO SPs in the transfer from *S. cerevisiae* to *K. phaffii* are rare, although Weissenborn and co-workers report successful transfer of systems between the two host organisms, albeit with some differences (Püllman et al. 2021). In one case, for example, the *Sce*-Invertase 2 SP was shown to be an effective SP for the production of the UPO from *Thielavia terrestris* (*Tte*UPO) in *K. phaffii*, but not in *S. cerevisiae.* Alcalde and co-workers reported that attempted expression of the native *Aae*UPO (presumably containing native SP and UPO enzyme sequence) from *A. aegerita* in both *S. cerevisiae* and *K. phaffii* resulted in poor protein production (Molina-Espeja et al. 2015). The performance of the native SP in the expression of variant enzyme *Aae*UPO-PaDa-I was not reported for either host strain, however the results in our present report suggest that, among the SPs studied, the n-SP is the superior SP for r*Aae*UPO-PaDa-I-H production in *K. phaffii*, but not in *S. cerevisiae.*

One factor that may operate in the effective secretion of the UPOs in the different hosts is the sequence of the SP peptidase cleavage site in the SP. It has previously been suggested that this site follows a conserved sequence of A-X-A in the SP (Perlman and Halvorson 1983), which would be predicted to be A^21^YA in the n-SP sequence, replaced by ANA and DYA in the 1C1-SP- and PaDa-I-SP sequences, respectively. We have previously suggested that, in *S. cerevisiae*, the Y22N mutation in the 1C1-SP may improve secretion of r*Aae*UPO-PaDa-I-H by relieving steric hindrance near the cleavage site for the signal peptidase in that organism. However, it may be that the substrate specificity of the signal peptidase in *K. phaffii* is sufficiently different to exhibit a preference for a tyrosine residue in this position, partially explaining the difference in SP effects on r*Aae*UPO-PaDa-I-H production between the two organisms.

Another possible factor may be the interaction of the SPs with the signal recognition particle (SRP), the ribonucleoprotein complex in the cytosol that interacts with nascent proteins and directs their translocation to the ER (Akopian et al. 2013). Each of the SPs used in this study differs in amino acid sequence in the region that is thought to be important for interaction with the SRP and more specifically the ‘M-domain’ of the SRP54 subunit of that complex (Egea et al. 2008). In order for the n-SP to outperform the engineered SPs, it is possible that more favourable interactions are made between the n-SP and the M-domain of SRP54 of *K. phaffii* than that of *S. cerevisiae.* A comparison of the sequences of the SRP54 subunits in *A. aegerita*, *S. cerevisiae* and *K. phaffii* (Figure S8) suggests that there is a slightly higher degree of identity between the SRP54 sequences of *A. aegerita* and *K. phaffi* (51.1%) than between *A. aegerita* and *S. cerevisiae* (47.9%). However, any effect of these differences in sequence on interactions between the SRPs and the r*Aae*UPO-PaDa-I-H SPs on protein production in the different yeast strains presented herein would require detailed structural analysis of the interactions between substituted amino acids and the M-domains in each case.

## Conclusion

In this work we compared the production of the UPO variant r*Aae*UPO-PaDa-I-H in *K. phaffii* using constructs with different SPs: the native sequence (n-SP) from *Agrocybe aegerita*, the variant PaDa-I-SP developed by Alcalde and coworkers (Molina-Espeja et al. 2014, 2015), and the 1C1-SP developed in our laboratory (Camboni et al. 2025). The experiments showed that r*Aae*UPO-PaDa-I-H production using the n-SP/*Aae*UPO-PaDa-I-H construct outperformed those of PaDa-I-SP/r*Aae*UPO-PaDa-I-H and 1C1/ r*Aae*UPO-PaDa-I-H. These results contrast with the comparative performance of each gene construct when expressed in *S. cerevisiae*, for which the 1C1/r*Aae*UPO-PaDa-I-H construct was superior to the other two (Camboni et al. 2025). These findings suggest that optimal SPs developed for one host organism might not readily transferable to another species, highlighting the importance in achieving an optimal balance between the SP, the target enzyme, and the host organism for optimal functional secretion of target proteins. In addition, the concentration of r*Aae*UPO-PaDa-I-H achieved in *K. phaffii* using the n-SP (365 mg L^−1^) also appears to exceed the highest concentrations of *Aae*UPO-PaDa-I previously reported in this host, which were 217 mg L^−1^ from fed-batch fermentation (Molina-Espeja et al. 2015), and 290 mg L^−1^, from pilot-scale fermentation (Tonin et al. 2021), suggesting an alternative approach to optimal enzyme production for this important target.

## Supplementary Information

Below is the link to the electronic supplementary material.

Supplementary Material 1.

## Data Availability

The datasets supporting the conclusions of this article are included within the article.
