# Supplementary material for: Enhanced secretion of an Agrocybe aegerita peroxygenase variant in K. phaffii using the native signal peptide
Source: AMB Express. 2026 Apr 1;16:53. doi: 10.1186/s13568-026-02049-x (PMC13168419; doi:10.1186/s13568-026-02049-x)
Supplement: Supplementary file 1 — Supplementary Material 1. [file 13568_2026_2049_MOESM1_ESM.docx]

**Enhanced secretion of an *Agrocybe aegerita* Peroxygenase variant in *K. phaffii* using the native signal peptide**

Ginevra Camboni,^a,b^ Rebecca Preece,^b^ Katy A.S. Cornish,^a,b^ Jared Cartwright^b^* and Gideon Grogan^a^*

Departments of Chemistry^a^ and Biology,^b^ University of York, Heslington, York YO10 5DD U.K.
jared.cartwright@york.ac.uk; gideon.grogan@york.ac.uk

**Supplementary File**

**Table S1. BMGY** (Buffered glycerol complex medium) prepared for gene expression in *K. phaffii*. Biotin was added fresh, before inoculation of colonies.

| **BMGY** (20 mL) | |
| --- | --- |
| YEP (Yeast extract and peptone) | 14 mL |
| Potassium phosphate | 2 mL |
| 10X YNB (Yeast nitrogen base) | 2 mL |
| 10% Glycerol | 2 mL |
| 500X Biotin | 40 µL |

**Table S2. BMMY (**Buffered methanol complex medium) prepared for gene expression in *K. phaffii*. Biotin was added fresh, before inoculation of colonies.

| **BMMY** (25 mL) | |
| --- | --- |
| YEP (Yeast extract and peptone) | 17.5 mL |
| Potassium phosphate | 2.5 mL |
| 10X YNB (Yeast nitrogen base) | 2.5 mL |
| 5% Methanol | 2.5 mL |
| 500X Biotin | 50 µL |

**Table S3.** **YPD (**Yeast extract, peptone and glucose medium) recipe.

| **YPD** (1 L) | |
| --- | --- |
| YPD extract | 10 g |
| dd H_2_O | 900 mL |
| Glucose (20% w/v)^1^ | 100 mL |

^1^The solution was autoclaved and afterwards the glucose (sterile) was added to the mix.

**
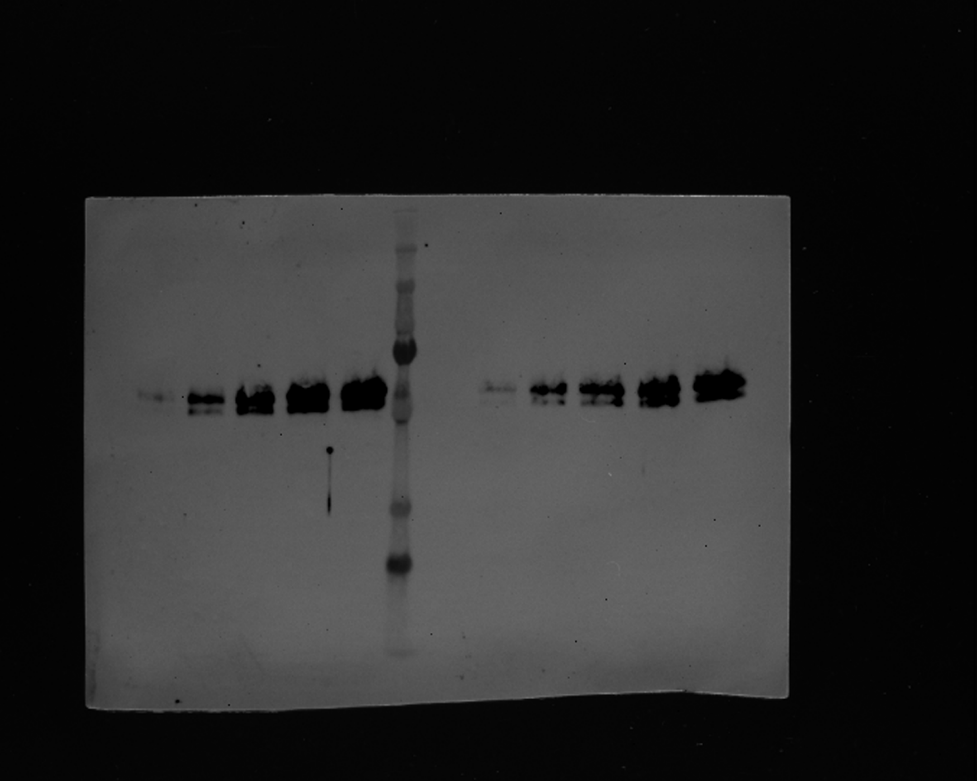
**

**Figure S1.** Uncropped Western Blot for Manuscript **Figure 3A (i).**


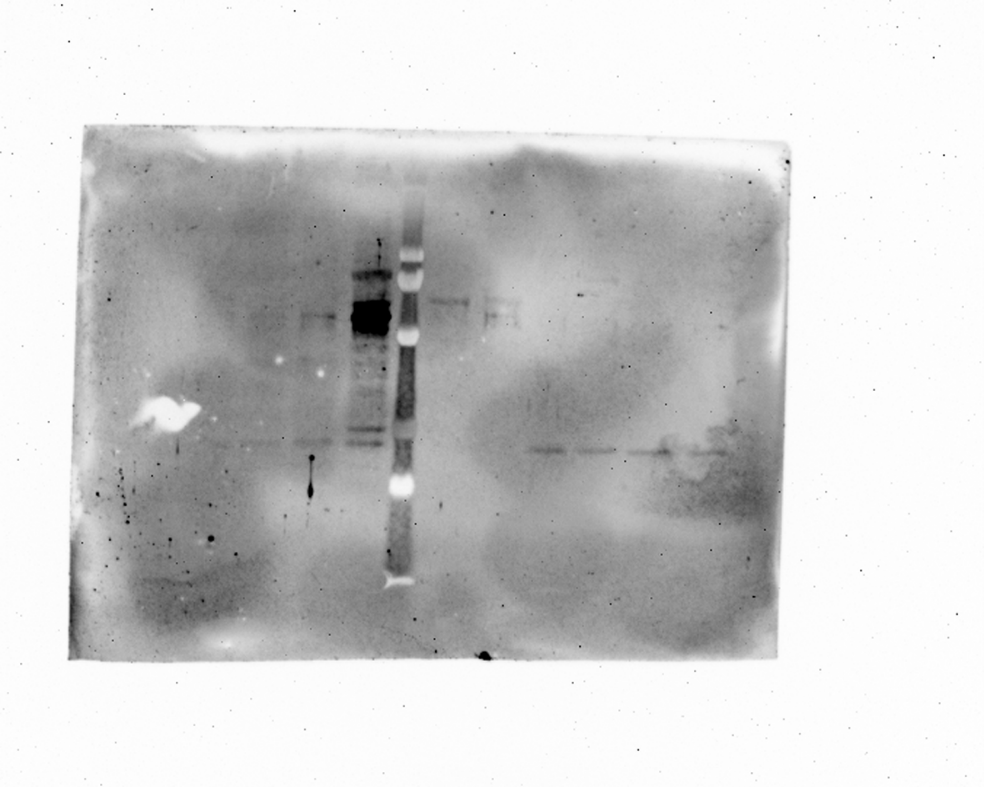


**Figure S2**. Uncropped Western Blot for Manuscript **Figure 3A (ii).**

**
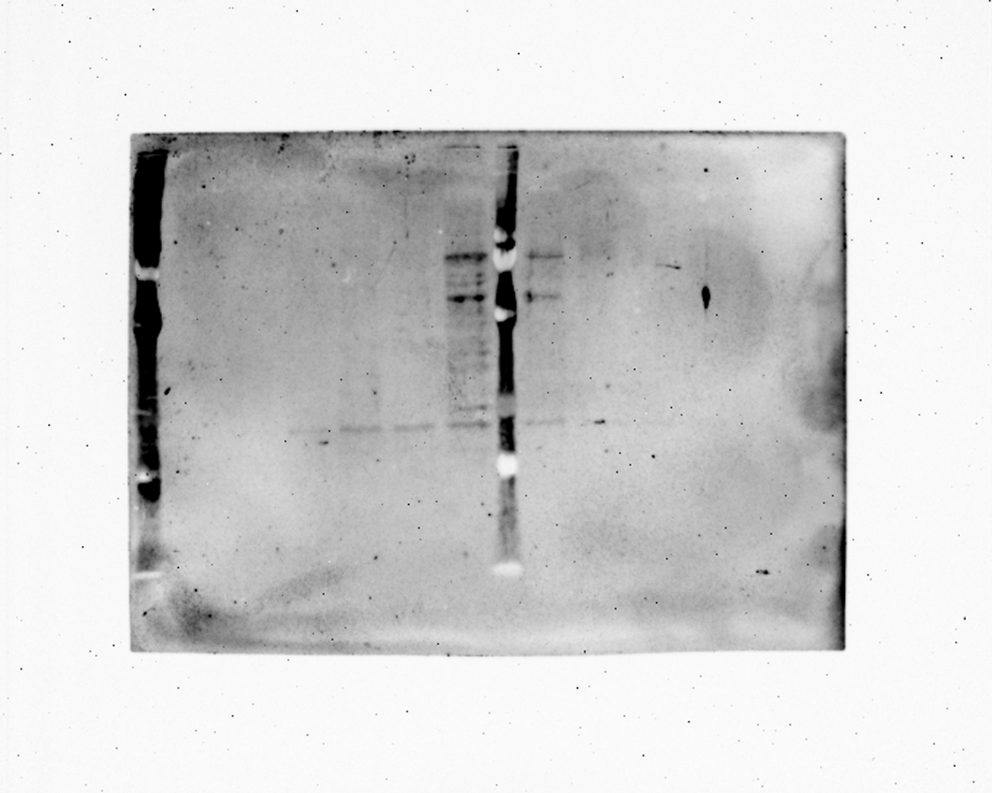
**

**Figure S3**. Uncropped Western Blot for Manuscript **Figure 3A (iii).**


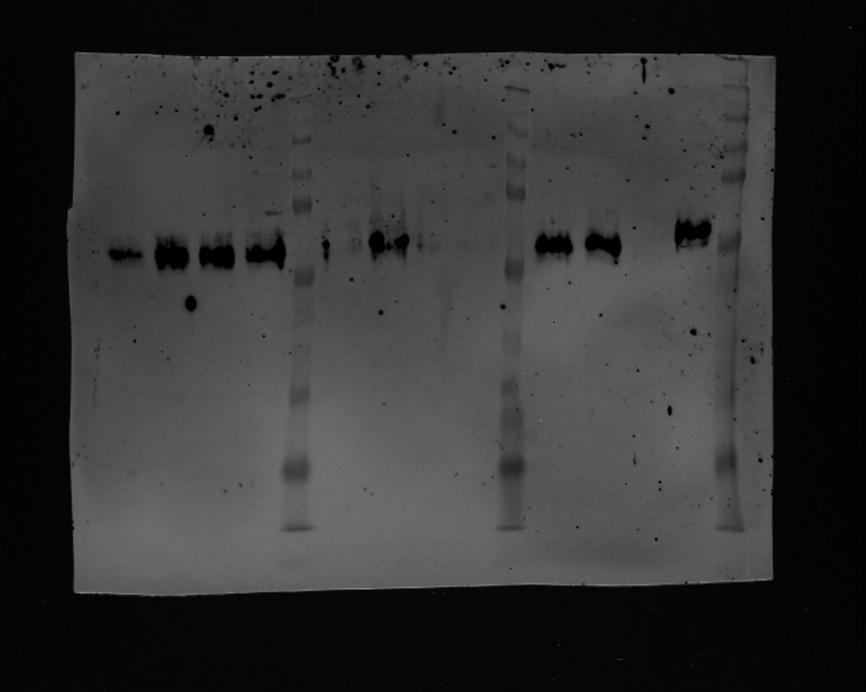


**Figure S4**. Uncropped Western Blot for Manuscript **Figure 4A.**

**
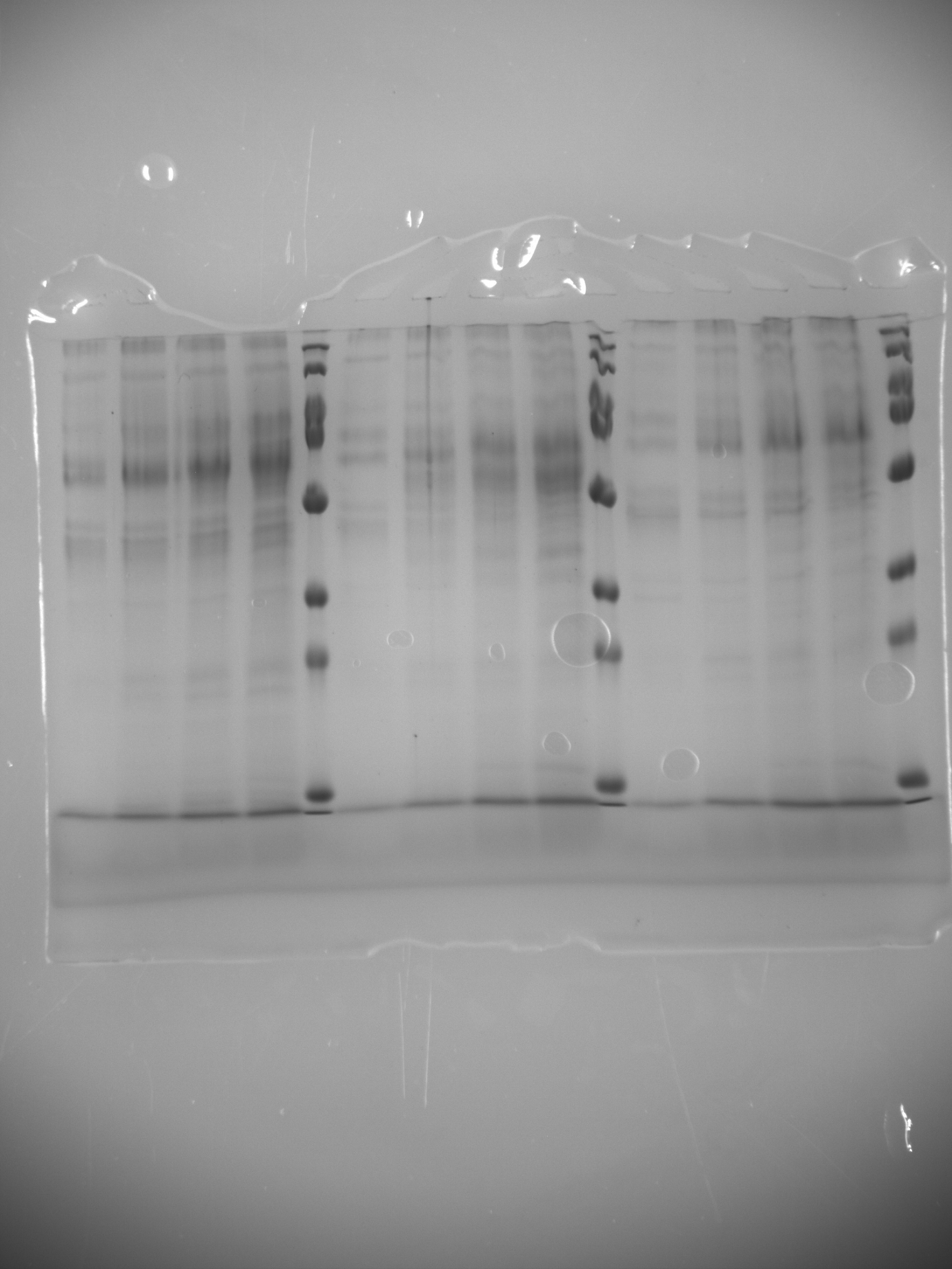
**

**Figure S5**. Uncropped SDS-PAGE gel for Manuscript **Figure 5A.**

**
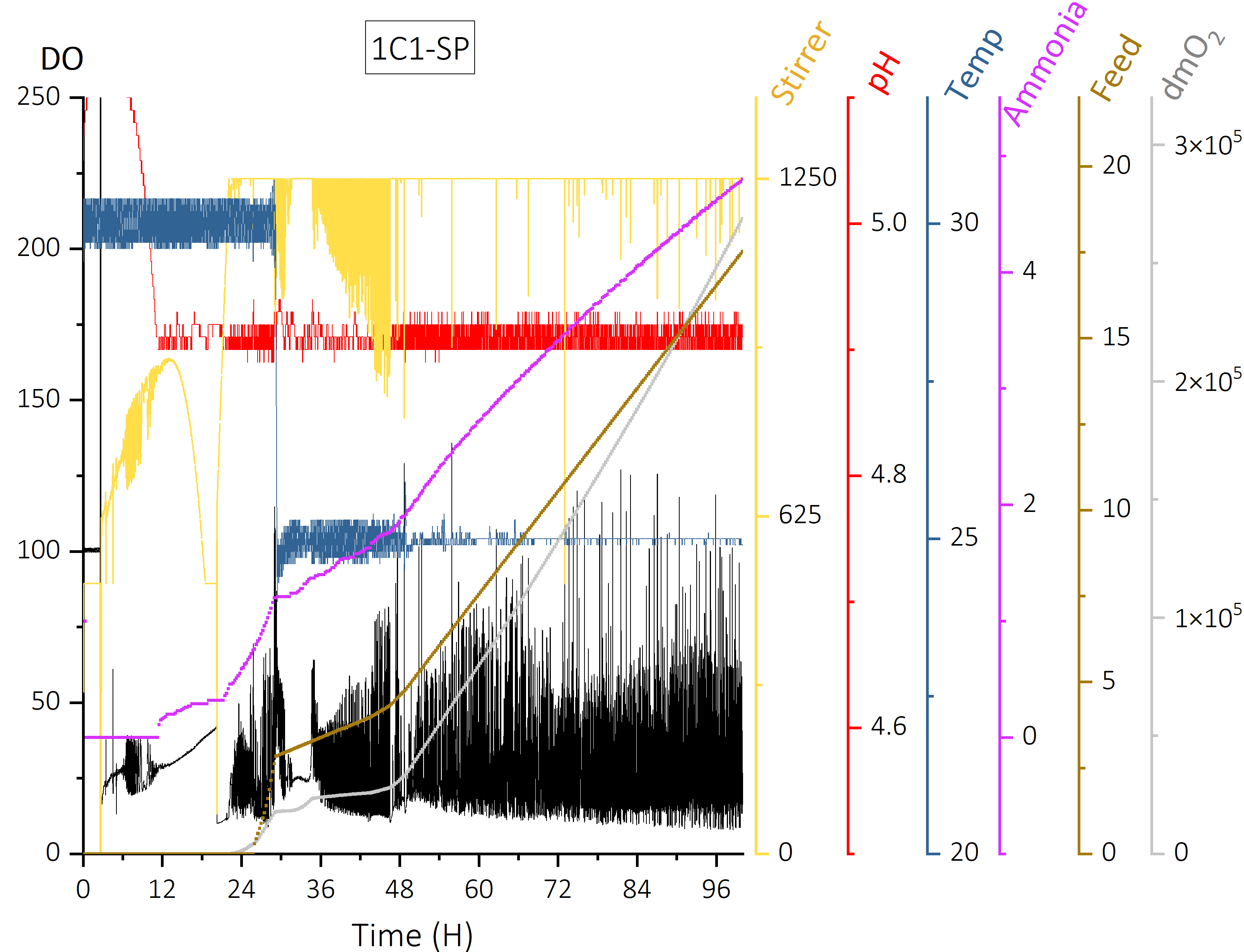
**

**Figure S6A**. Graph showing the progress of fermentation of cells using 1C1-SP. Plot lines follow the dissolved oxygen (DO, black, range 0-250%); stirrer speed (yellow, 300-1250 rpm); pH (red, 4.75-5); temperature (blue, 25-30°C); ammonia addition (purple, mL) to balance the pH; feeding solutions addition (brown, mL) and the dmO_2_ (dose monitor O_2_,grey, L), indicating the level of supplemental oxygen added into the vessel. Note: axes scales were adapted for visual consistency.


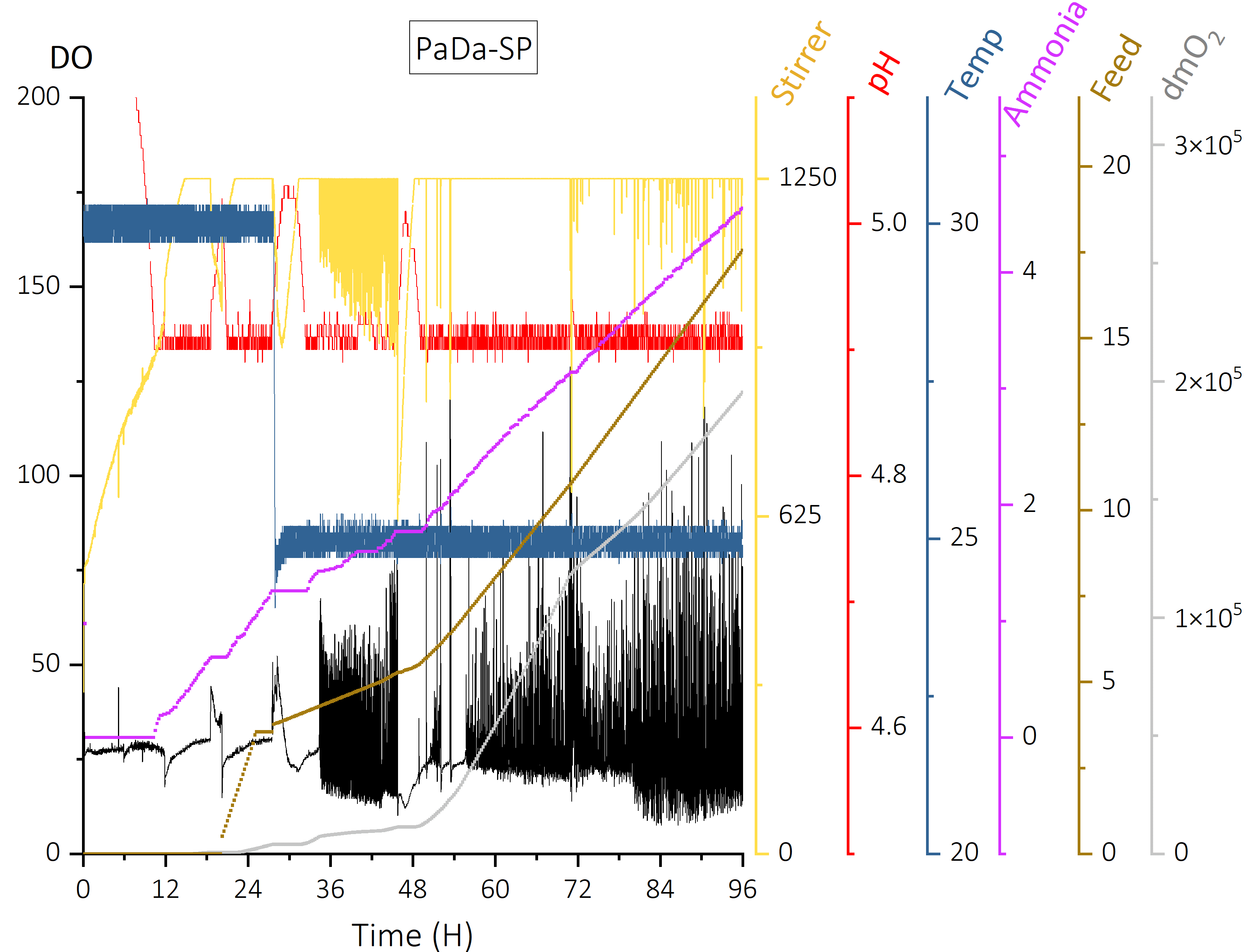


**Figure S6B**. Graph showing the progress of fermentation of cells using PaDa-SP. Plot lines follow the dissolved oxygen (DO, black, range 0-200%); stirrer speed (yellow, 300-1250 rpm); pH (red, 4.75-5); temperature (blue, 25-30°C); ammonia addition (purple, mL) to balance the pH; feeding solutions addition (brown, mL) and the dmO_2_ (dose monitor O_2_ ,grey, L), indicating the level of supplemental oxygen added into the vessel. Note: axes scales were adapted for visual consistency.

**
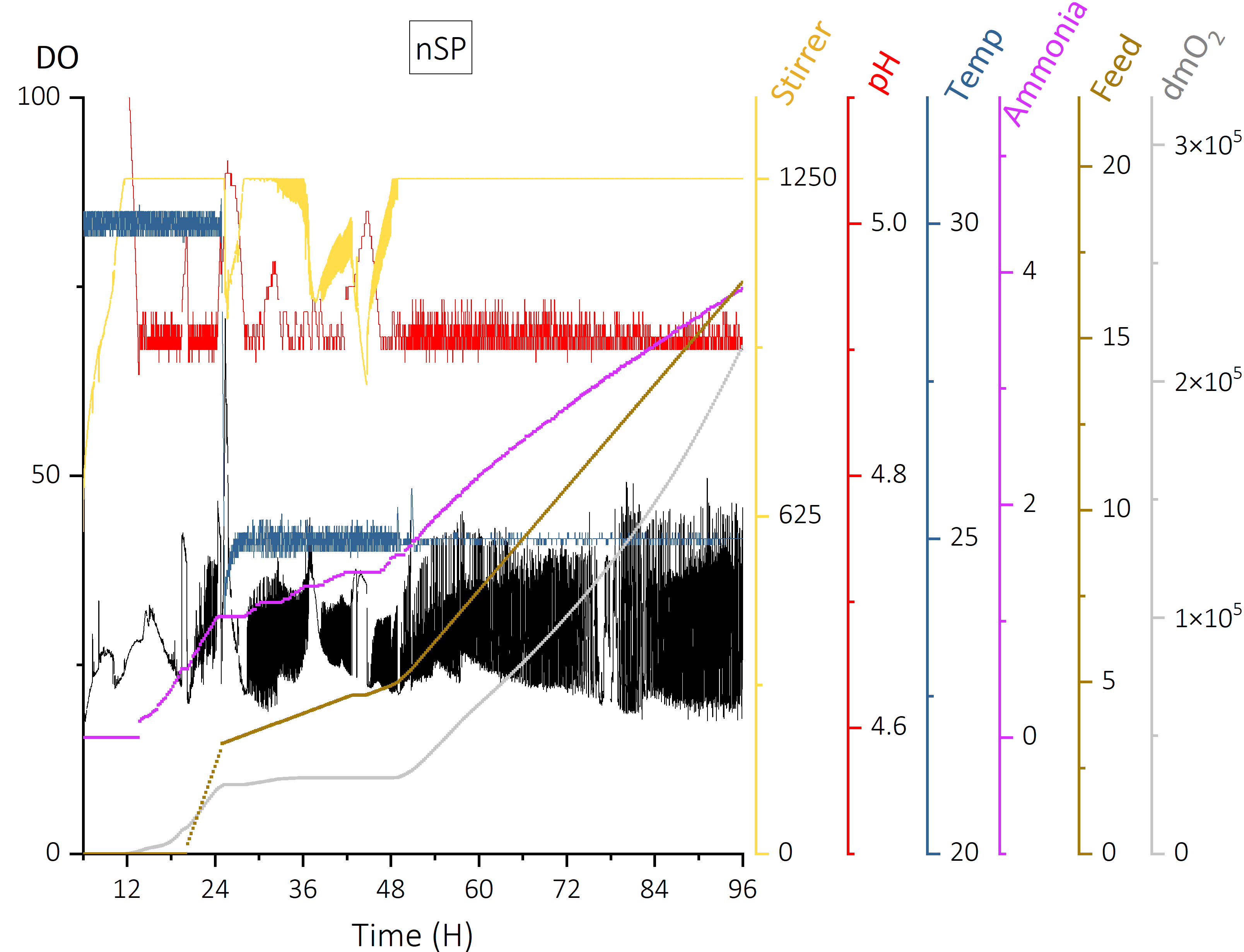
**

**Figure S6C**. Graph showing the progress of fermentation of cells using n-SP. Plot lines follow the dissolved oxygen (DO, black, range 0-100%); stirrer speed (yellow, 300-1250 rpm); pH (red, 4.75-5); temperature (blue, 25-30°C); ammonia addition (purple, mL) to balance the pH; feeding solutions addition (brown, mL) and the dmO_2_ (dose monitor O_2_, grey, L), indicating the level of supplemental oxygen added into the vessel. Note: axes scales were adapted for visual consistency.

**Table S4**. Wet-cell weight measured from 2 mL samples collected during fermentations of cells carrying named constructs.

|  | T = 0 h | T = 24 h | T = 48 h | T = 72 h (when harvested) |
| --- | --- | --- | --- | --- |
| **pPICZB/n-SP/r*Aae*UPO-PaDa-I-H** | 325.4 mg | 329.7 mg | 490.9 mg | 553.6 mg |
| **PaDa-I-SP/r*Aae*UPO-PaDa-I-H** | 294.1 mg | 333.3 mg | 502.1 mg | 604.1 mg |
| **pPICZB/1C1-SP/r*Aae*UPO-PaDa-I-H** | 338 mg | 408.5 mg | 555.7 mg | 677 mg |

**
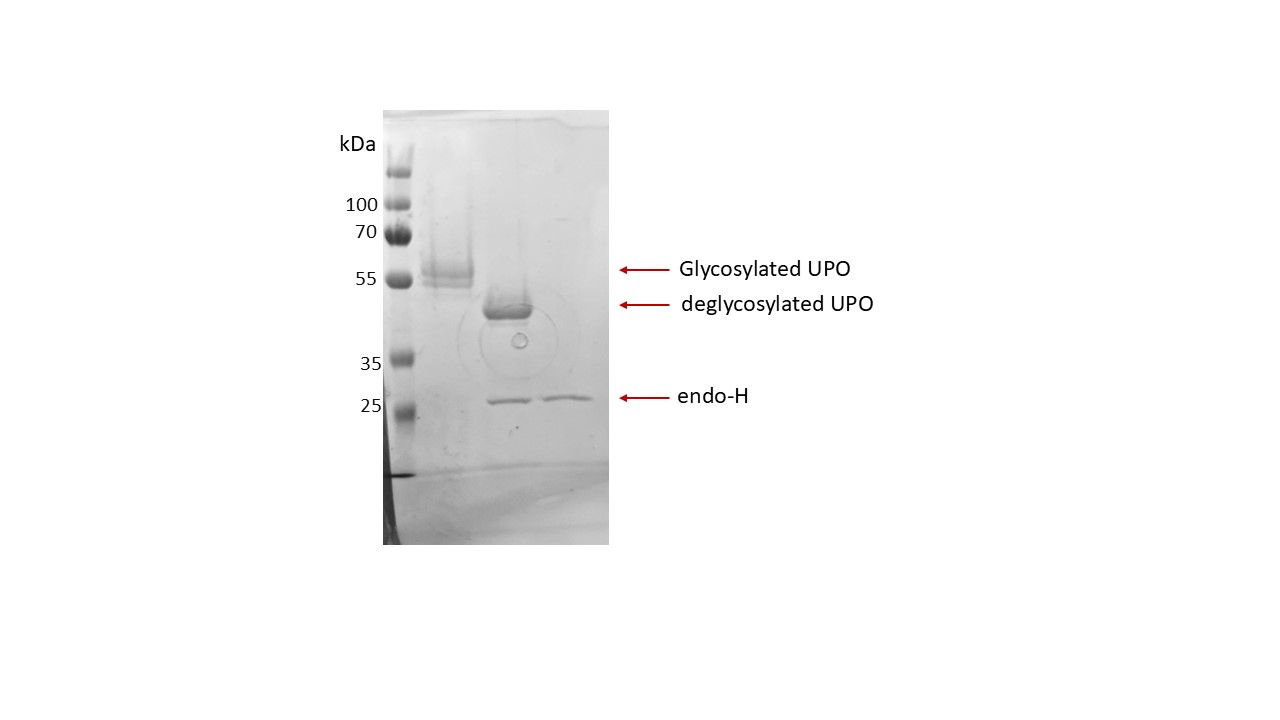
**

**Figure S7**. SDS-PAGE gel showing glycosylated r*Aae*UPO-PaDa-I-H (UPO) produced with glycosylation, and after deglycosylation using Endo-H.

KphSRP54 MVLADLGRRINNAVGNVTKSNVVDADV-ISNMLKEICNALLESDVNIKLVAQLREKIRKQ

SceSRP54 MVLADLGKRINSAVNNAISNTQDDFTTSVDVMLKGIVTALLESDVNIALVSKLRNNIRSQ

AaeSRP54 MVLADLGRKLNAALSALNRAPVVDEKV-LDATLKEITAALLESDVNVKLVASLRQKVKAK

*******:::* *:. * . :. ** * ********: **:.**:::: :

KphSRP54 IDA--EDKP-----GINKKKLIQKVVFDELVKLVDCN--EAELFKPKKKQTNVIMMVGLQ

SceSRP54 LLS--ENRSEKSTTNAQTKKLIQKTVFDELCKLVTCEGSEEKAFVPKKRKTNIIMFVGLQ

AaeSRP54 VKATFEGNADKSK-EANRKNVIQKAVFDELVALVDPG---VEPYKPKKGQSNVIMAVGLQ

: : *... : *::***.***** ** : : *** ::*:** ****

KphSRP54 GAGKTTTCTKLAVYYQRRGFKVGMVCGDTFRAGAFDQLKQNATKAKIPYYGSYTETDPVK

SceSRP54 GSGKTTSCTKLAVYYSKRGFKVGLVCADTFRAGAFDQLKQNAIRARIPFYGSYTETDPAK

AaeSRP54 GNGKTTTCTKLAVHYQKRGFKSAIVCADTFRAGAFDQTRQSATKAKVAYFGSYTETDPVS

* ****:******:*.:**** .:**.********** :*.* :*::.::********..

KphSRP54 VTFDGVEEFRKEKFEIIIVDTSGRHRQEEDLFEEMVQIGKAIKPNQTIMVLDASIGQSAE

SceSRP54 VAEEGINKFKKEKFDIIIVDTSGRHHQEEELFQEMIEISNVIKPNQTIMVLDASIGQAAE

AaeSRP54 IAAQGVAKFKKERFDVIIVDTSGRHKQESELFEEMVQIGEAVRPNMTVLILDASIGQAAE

:: :*: :*:**:*::*********:**.:**:**::*.:.::** *:::*******:**

KphSRP54 SQSKAFKESSDFGAIIITKMDSNSKGGGALSAIAATNTPVAFIATGEHIQNFEKFSGRGF

SceSRP54 QQSKAFKESSDFGAIILTKMDGHARGGGAISAVAATNTPIIFIGTGEHIHDLEKFSPKSF

AaeSRP54 AQSRAFKDSADFGAIIVTKMDGHAKGGGAISAVAATKTPIIFLGVGEHLHDLDRFSPEPF

**:***:*:******:****.:::****:**:***:**: *:..***::::::** . *

KphSRP54 ISKLLGIGDIEGLMEHVQSMN----LDQGDTIKNFKEGKFTLQDFQTQLNNIMKMGPLSK

SceSRP54 ISKLLGIGDIESLFEQLQTVSNK--EDAKATMENIQKGKFTLLDFKKQMQTIMKMGPLSN

AaeSRP54 ISKLLGLGDVQGLMEHMQDLATQNPDKQKEMAKKLEEGKLSIRDWREQISNVMNMGPISK

******:**::.*:*::* : . :::::**::: *:: *:..:*:***:*:

KphSRP54 LAQMLPGGMGQLMGQVGEEEASKRLKRMIYIMDSMTKQELASDGRLFIDQPS--------

SceSRP54 IAQMIPG-MSNMMNQVGEEETSQKMKKMVYVLDSMTKEELESDGRMFIEEPT--------

AaeSRP54 IASMIPG-LPQDLLQGSDEEGSLRMKRMIYITDSMTASELDSDGSPFMELEKDGKPVGLT

:*.*:** : : : * .:** * ::*:*:*: **** .** *** *:: .

KphSRP54 -RMVRVARGSGTSVTEVELVLLQQKMMARMALQS----------KNMMSGAGGP----GG

SceSRP54 -RMVRVAKGSGTSVFEVEMILMQQQMMARMAQTA----------TQQQPGAPGANARMPG

AaeSRP54 WRVTRVARGSGTSVREVEELLCQYRMMANMAKQAGGKNGWLQAMQKMQAAAGGKGRGAGG

*:.***:****** *** :* * :***.** : : ..* * *

KphSRP54 MA----------------S-KMNPANMRRAMQQMQSNPGMMDNMMNMFGG-AGGAGGAGM

SceSRP54 MPNMPGMPNMPGMPNMPGMPKVTPQMMQQAQQKLKQNPGLMQNMMNMFGGGMGGGMGGGM

AaeSRP54 MP-TP--------AQIQAMQRSMPPGMLQQMQKQLRNGGGMQEMMKAMMQ-SQGGDQLDM

*. : * * : *: * * *::**: : *. .*

KphSRP54 PDMQEMMKQMSSGQMKMPSQQ----------EMMSMMKQFGMG------

SceSRP54 PDMNEMMKMMQDPQMQQ------------------MAKQFGMG------

AaeSRP54 EEMQRMMQQMGGGLGGLGGLGGLGGLGGLGGGMADMLKMMGMGGAGGGR

:*:.**: * . * * :***

**Figure S8**. Sequence alignment of Signal Recognition Particle (SRP) SRP54 subunits from *K. phaffii* (ANZ75037.1), *S. cerevisiae* (NP_015413.1) and *A. aegerita* (CAA7271651.1). Sequence identities are 51.1% between AaeSRP54 and KphSRP54 and 47.9% between AaeSRP54 and SceSRP54.
